# Extracellular vesicles derived from immobilization‐induced atrophic muscle of male mice contain distinct microRNA profiles and impact mRNA profiles in brain neurons

**DOI:** 10.14814/phy2.71052

**Published:** 2026-08-13

**Authors:** Noriaki Kawanishi, Shuichi Machida, Katsuhiko Suzuki

**Affiliations:** ^1^ Faculty of Advanced Engineering Chiba Institute of Technology Narashino Japan; ^2^ Research Organization for Nano & Life Innovation Waseda University Shinjuku‐ku Tokyo Japan; ^3^ Graduate School of Health and Sports Science Juntendo University Inzai Chiba Japan; ^4^ Faculty of Sport Sciences Waseda University Tokorozawa Saitama Japan

**Keywords:** brain neurons, exosomes, extracellular vesicles, microRNA, muscle atrophy

## Abstract

Muscle atrophy caused by inactivity leads to declines in multiple physiological functions, including brain function. Although skeletal muscle is known to secrete extracellular vesicles (EVs) such as exosomes, how inactivity‐induced muscle atrophy alters the properties and functions of these EVs remains unclear. In this study, we investigated the effects of cast immobilization–induced muscle atrophy on the microRNA (miRNA) profiles of skeletal muscle‐derived EVs in mice, as well as their impact on transcriptome changes in brain neurons. Muscle atrophy induced by cast immobilization significantly altered the microRNA profiles of skeletal muscle‐derived EVs, with 25 microRNAs upregulated and 2 microRNAs downregulated compared with controls. Moreover, treatment of brain neurons with EVs derived from atrophic skeletal muscle markedly changed neuronal mRNA expression profiles. Gene Ontology (GO) analysis revealed that upregulated mRNAs in EV‐treated neurons were enriched in genes involved in the positive regulation of programmed cell death, including apoptosis. Consistently, Kyoto Encyclopedia of Genes and Genomes (KEGG) pathway analysis demonstrated activation of apoptosis‐related signaling pathways in brain neurons. These findings suggest that muscle atrophy–induced alterations in skeletal muscle‐derived EVs may contribute to brain dysfunction by promoting apoptotic processes in brain neurons.

## 1 INTRODUCTION

Aging and inactivity decrease skeletal muscle mass and strength, thereby increasing the risk of developing sarcopenia and skeletal muscle atrophy (Frontera & Ochala, 2015). Importantly, a recent meta‐analysis revealed that the severity of sarcopenia (age‐related muscle loss) was closely related to cognitive impairment (Amini et al., 2024). Therefore, loss of skeletal muscle mass may be a risk factor for neurodegenerative diseases such as Alzheimer's disease. Recent studies have reported that skeletal muscle atrophy caused by cast immobilization leads to memory impairments in mice (Nagase & Tohda, 2021). However, further elucidation of the molecular mechanisms underlying cognitive decline caused by inactivity‐related muscle atrophy is needed for the development of effective therapies for neurodegenerative diseases such as Alzheimer's disease.

Skeletal muscles secrete many bioactive substances, such as hormones, growth factors, and cytokines. Exercise stimulates the secretion of humoral factors such as irisin, cathepsin B, and interleukin (IL)‐6 from skeletal muscle tissue, which pass through the blood–brain barrier (BBB) and induce the proliferation of nerve cells in the brain, thereby contributing to improved brain function (Pedersen, 2019). Skeletal muscle atrophy may alter the production and release of inter‐tissue communication factors. However, it has been shown that inter‐tissue communication factors are produced from atrophic skeletal muscle tissue due to aging and inactivity. It has been reported that hemopexin is secreted into the culture medium of atrophic skeletal muscle tissue by cast immobilization, and the administration of this humoral factor into mice causes memory dysfunction (Iki & Tohda, 2024). Therefore, inter‐tissue communication factors secreted by atrophied skeletal muscles may play an important role in the development of disuse‐induced muscle atrophy‐related cognitive impairment.

Extracellular vesicles including exosomes and microvesicles are secreted by various cell types and are known to have various pathophysiological functions (Kalluri & LeBleu, 2020). Exosomes contain many types of cell‐derived microRNAs (miRNAs), proteins, and lipids. Exosomes secreted from resident cells in several tissues function as endocrine factors that are transported through blood circulation. It has been shown that extracellular vesicles can cross the blood–brain barrier (BBB) (Li et al., 2022), suggesting that they may be an important factor in the regulation of the central nervous system by peripheral tissue.

Extracellular vesicles are also secreted from skeletal muscle tissue. Our and other studies have suggested that the stimulation of skeletal muscle contraction by exercise changes the microRNA expression profile of extracellular vesicles secreted from the skeletal muscle (Frühbeis et al., 2015; Kawanishi et al., 2023). In contrast, aging‐ and disuse‐induced muscle atrophy has been found to change the microRNAs composition in circulating extracellular vesicles. Xhuti et al. reported that elderly subjects with progressive muscle atrophy have lower expression levels of several microRNAs contained in circulating extracellular vesicles compared to young subjects, and that the expression levels of these microRNAs are reduced in extracellular vesicles secreted from aged myotubes after repeated passages (Xhuti et al., 2023). In disuse‐induced muscle atrophy model mice, tail suspension alters the microRNA expression profile of circulating extracellular vesicles in mice (Hayasaka et al., 2021). Furthermore, Parker et al. reported that lower‐limb immobilization alters the microRNA expression profile of extracellular vesicles contained in fibro‐adipogenic progenitor cells localized in skeletal muscle (Parker et al., 2022). Recent studies have suggested that extracellular vesicles secreted from skeletal muscle not only affect muscle tissue itself but also influence distant tissues. For example, administration of extracellular vesicles derived from starvation‐induced atrophied muscle fibers has been reported to inhibit the differentiation of muscle satellite cells in mice (Shao et al., 2022). Furthermore, administration of extracellular vesicles secreted from atrophied skeletal muscle of aged mice to young mice decreased bone mineral density and bone strength by suppressing bone formation (Shao et al., 2026). In addition, extracellular vesicles secreted from denervation‐induced atrophied skeletal muscle were reported to exacerbate renal fibrosis in mice with renal injury (Jiang et al., 2025). Collectively, these findings suggest that alterations in the properties of extracellular vesicles secreted from atrophic skeletal muscle induced by inactivity may affect the function of distant tissues.

Although it is important to examine changes in the microRNA profile of extracellular vesicles to evaluate the role of microRNAs in skeletal muscle‐derived extracellular vesicle‐mediated multitissue interactions, changes in the microRNA profiles of extracellular vesicles secreted from atrophic skeletal muscle itself in response to cast immobilization‐induced inactivity have not been examined yet. Therefore, the aim of this study is to investigate the effect of muscle atrophy induced by cast immobilization on the microRNA profiles of skeletal muscle‐derived extracellular vesicles in mice. Additionally, this study investigated the effects of extracellular vesicles (EV) secreted from atrophied skeletal muscles on altered transcriptome profiles in brain neurons.

## 2 MATERIALS AND METHODS

### 2.1 Animals

Ten‐weeks‐old male C57BL/6 mice were obtained from Japan SLC (Shizuoka, Japan). Mice were acclimated for 2 weeks in a controlled environment maintained at 24°C with a 12‐h light/dark cycle and provided food (Laboratory Rodent Diet, catalog no. 5001; LabDiet) and water ad libitum. At 12 weeks of age, mice were randomly assigned to either the cast immobilization group or the control group (*n* = 6/each group) for subsequent experiments.

### 2.2 Cast immobilization protocol

Hindlimb immobilization was performed in the cast immobilization group, as previously described (Kawanishi et al., 2018). Briefly, mice were lightly anesthetized with isoflurane (Abbott, Tokyo, Japan) prior to the application of casting materials. Both hindlimbs were fixed in a shortened position using casting tapes (Scotchcast Plus‐J; 3 M Health Care, St. Paul, MN, USA). Following immobilization, mice were housed individually with ad libitum access to water and standard food. Cast integrity was monitored daily and repaired as needed.

After 14 days of immobilization, mice were euthanized using cervical dislocation under anesthesia. Casts were removed under isoflurane anesthesia, and blood was drawn via transcardial perfusion with 20 mL of phosphate‐buffered saline (PBS) using a peristaltic pump. Hindlimb skeletal muscles, including the soleus, plantaris, gastrocnemius, and quadriceps femoris, were carefully dissected and weighed.

### 2.3 Incubation of skeletal muscle and isolation of extracellular vesicles

Skeletal muscle‐derived extracellular vesicles were isolated as described in previous study (Ma et al., 2023). Briefly, gastrocnemius was rinsed, cut into pieces and cultured in 6‐well dishes in 2‐mL DMEM containing 10% EV‐free fetal bovine serum (FBS) (Exosome‐depleted FBS Media Supplement, catalog no. EXO‐FBS‐50A‐1; System Biosciences, CA, USA). After 24 h of culture, the supernatant was collected, and the cell debris was removed by centrifugation. Extracellular vesicles present in the supernatant were isolated using the Total Exosome Isolation Reagent (catalog no. 4478359; Thermo Fisher Scientific, Waltham, MA, USA). Briefly, 2 mL of the supernatant was mixed with 1 mL of Total Exosome Isolation Reagent and incubated at 4°C overnight. The mixture was then centrifuged at 10,000 × *g* at 4°C for 60 min. The resulting EVs pellet was resuspended in 200 μL of PBS and stored at −80°C until further analysis.

### 2.4 Measurement of extracellular vesicle particle size

Particle size measurement was performed using Interferometric Light Microscopy on a VIDEO DROP (Corning, NY, USA). The EVs were diluted 1:100 in PBS, and the diluted samples were analyzed by two separate injections. For each injection, a 60‐s video was recorded.

### 2.5 Western blotting of extracellular vesicle marker

Skeletal muscle‐derived extracellular vesicles were mixed with 4 × Laemmli sample buffer (catalog no. 1610747; BioRad, Richmond, CA, USA) and 5% 2‐mercaptoethanol and heat‐denatured for 5 min at 95°C. Reducing agents (2‐mercaptoethanol) were used only for the analysis of TSG101 and Alix. The mixed samples were resolved using 10% SDS‐polyacrylamide gel electrophoresis and transferred to a polyvinylidene difluoride (PVDF) membrane. The membrane was blocked with PVDF Blocking Reagent (catalog no. NYPBR01; TOYOBO, Osaka, Japan) at 25°C for 1 h and incubated overnight at 4°C with an appropriate primary antibody (1:5000 dilution for CD9 [catalog no. 98327; Cell Signaling Technology, Beverly, MA, USA], 1:5000 dilution for CD81 [catalog no. 10037; Cell Signaling Technology], 1:5000 dilution for Alix [catalog no. 92880; Cell Signaling Technology]), 1:5000 dilution for TSG101 (catalog no. 72312; Cell Signaling Technology). The membrane was then incubated for 1 h at room temperature with horseradish peroxidase‐linked secondary antibody (1:10000 dilution for anti‐mouse IgG [7074; Cell Signaling Technology]). Chemiluminescence was developed using Clarity Western ECL Substrate (catalog no. 1705060; BioRad) and detected by a CCD camera (ChemiBOX Mi‐II 140CB, Biotools, Gunma, Japan).

### 2.6 RNA isolation from extracellular vesicles

Total RNA was extracted from the skeletal muscle–EVs using the Total Exosome RNA and Protein Isolation Kit (catalog no. 4478545; Thermo Fisher Scientific). Total RNA concentration was quantified using a Qubit 3 Fluorometer and a Qubit RNA High Sensitivity Assay Kit (catalog no. Q32852; Thermo Fisher Scientific).

### 2.7 Small RNA library preparation and sequencing

Small RNA libraries were prepared by using the QIAseq miRNA Library Kit and QIAseq miRNA NGS Index TF (catalog no. 331502, 331585; Qiagen, Hilden, Germany) according to the manufacturer's protocol. Briefly, small RNA‐seq libraries were constructed from 10 ng of RNA isolated from skeletal muscle‐derived extracellular vesicles. Library concentrations were quantified by using a Qubit 3 Fluorometer and the Qubit dsDNA Broad Range Assay Kit (catalog no. Q33265; Thermo Fisher Scientific). For sequencing, libraries were pooled at equimolar concentrations and performed to 100 bp single‐end sequencing on the Ion Torrent Ion GeneStudio S5 system (Thermo Fisher Scientific). Each small RNA library yielded over six million reads. Sequencing data have been deposited in the DNA Data Bank of Japan Sequence Read Archive (accession number: DRA020350).

Small RNA‐seq reads data were analyzed by using the GeneGlobe Data Analysis Center (Qiagen). Briefly, small RNA‐seq reads were mapped to microRNAs by using miRBase, and the different RNA transcripts, including miRNAs, were counted. The read counts of the microRNA species were normalized by adjusting to counts per one million. The miRNA species with more than 10 reads were included in the differential expression analysis. Differentially expressed miRNAs were defined as those with an absolute log2 fold change greater than 1 (corresponding to a 2‐fold change) and FDR < 0.01.

### 2.8 Incubation of skeletal muscle‐derived extracellular vesicles with brain neuron cells

Primary neuronal stem cells derived from the hippocampus of C57BL/6 mice were purchased from Thermo Fisher Scientific (Primary Mouse Hippocampal Neurons, catalog no. A15587) and cultured according to the manufacturer's instructions. Primary neural stem cells were incubated in poly‐D‐lysine‐coated 24‐well plates (1 × 10^5^/well) with Neurobasal Medium (catalog no. 21103049; Thermo Fisher Scientific) containing B‐27 Supplement (catalog no. 17504044; Thermo Fisher Scientific) and penicillin and streptomycin, in 5% CO_2_ at 37°C. After incubation for 14 days in the differentiation medium, the neuronal cells were used for experiments. After the neuron cell media was replaced with fresh EV‐free medium, the cells were treated with 2.5 × 10^8^ skeletal muscle‐derived extracellular vesicles from mice and cultured for 6 h. For nontreatment control conditions, neurons were cultured under identical experimental conditions without addition of EV preparations for the same incubation period used in EV‐treatment experiments. Following EV treatment, cells were lysed using QIAzol Lysis Reagent (catalog no. 79306; Qiagen), and lysates were stored at −80°C.

### 2.9 mRNA library preparation and sequencing

mRNA libraries were prepared by using the Ion AmpliSeq Transcriptome Mouse Gene Expression Kit and the Ion Xpress Barcode Adapters Kit (catalog no. A36554; Thermo Fisher Scientific), according to the manufacturer's protocols. Briefly, mRNA‐seq libraries were constructed from 10 ng of RNA isolated from brain neurons. Library concentrations were quantified using the Ion Library TaqMan Quantitation Kit (catalog no. 4468802; Thermo Fisher Scientific). For sequencing, libraries were pooled at equimolar concentrations and performed 150 bp single‐end sequencing on the Ion Torrent Ion GeneStudio S5 system.

The mRNA‐seq reads data were analyzed by using Torrent Suite Software (Version numbers 5.18; Thermo Fisher Scientific). The mRNA read counts were normalized to counts per one million. The mRNA species with more than 10 reads were included in the differential expression analysis. Differentially expressed mRNAs were defined as those with an absolute log2 fold change greater than 0.58 (corresponding to a 1.5‐fold change) and FDR <0.01.

Gene Ontology (GO) and Kyoto Encyclopedia of Genes and Genomes (KEGG) pathway enrichment analyses were conducted by using the DAVID Bioinformatics Resources (Version numbers 6.8; https://davidbioinformatics.nih.gov/) Enrichment significance for GO terms and KEGG pathways was assessed by using a *p*‐value (*p* < 0.05). In addition, pathways with 0.05 ≤ *p* < 0.10 were considered trend‐level enrichments and are discussed as exploratory observations.

### 2.10 Statistical analysis

Statistical analyses were performed by using GraphPad Prism version 7.0 (GraphPad Software Inc., CA, USA). The *p*‐value for the multiple comparison *t*‐test of the sequence data was corrected by using the false discovery rate. *p*‐values were adjusted for multiple comparisons using the Benjamini–Hochberg false discovery rate (FDR) correction method, and an FDR threshold of 1% was applied to determine statistical significance. Differences in muscle mass and EV number between the two groups were assessed using Student's *t*‐test, with statistical significance defined as *p* ≤ 0.05.

## 3 RESULTS

### 3.1 Body mass and tissue mass

There was no significant difference in the body mass between the two groups (Table 1). Skeletal muscle tissue mass of the soleus, plantaris, gastrocnemius, and quadriceps femoris muscles was lower in the cast immobilization group mice than in the control group (Table 1).

**TABLE 1:** Body mass and muscle mass in control mice and cast immobilization mice.

	Control (*n* = 6)	Cast immobilization (*n* = 6)	*p*‐Value
**Body mass (g)**			
Pre	24.2 ± 0.7	24.2 ± 0.5	
Post	25.0 ± 0.7	24.0 ± 0.4	
**Skeletal muscle mass (mg)**			
Soleus	9.4 ± 0.2	7.5 ± 0.2	<0.01
Plantaris	18.4 ± 0.6	15.7 ± 0.3	<0.01
Gastrocnemius	141.3 ± 3.7	118.9 ± 2.3	<0.01
Quadriceps muscle	222.3 ± 6.2	183.6 ± 3.7	<0.01
**Skeletal muscle mass/body mass (mg/g)**			
Soleus	0.37 ± 0.01	0.31 ± 0.01	<0.01
Plantaris	0.74 ± 0.01	0.65 ± 0.01	<0.01
Gastrocnemius	5.64 ± 0.03	4.95 ± 0.06	<0.01
Quadriceps muscle	8.89 ± 0.09	7.64 ± 0.05	<0.01

### 3.2 Characteristics of extracellular vesicles isolated from the culture medium of mouse skeletal muscle

**FIGURE 1:**
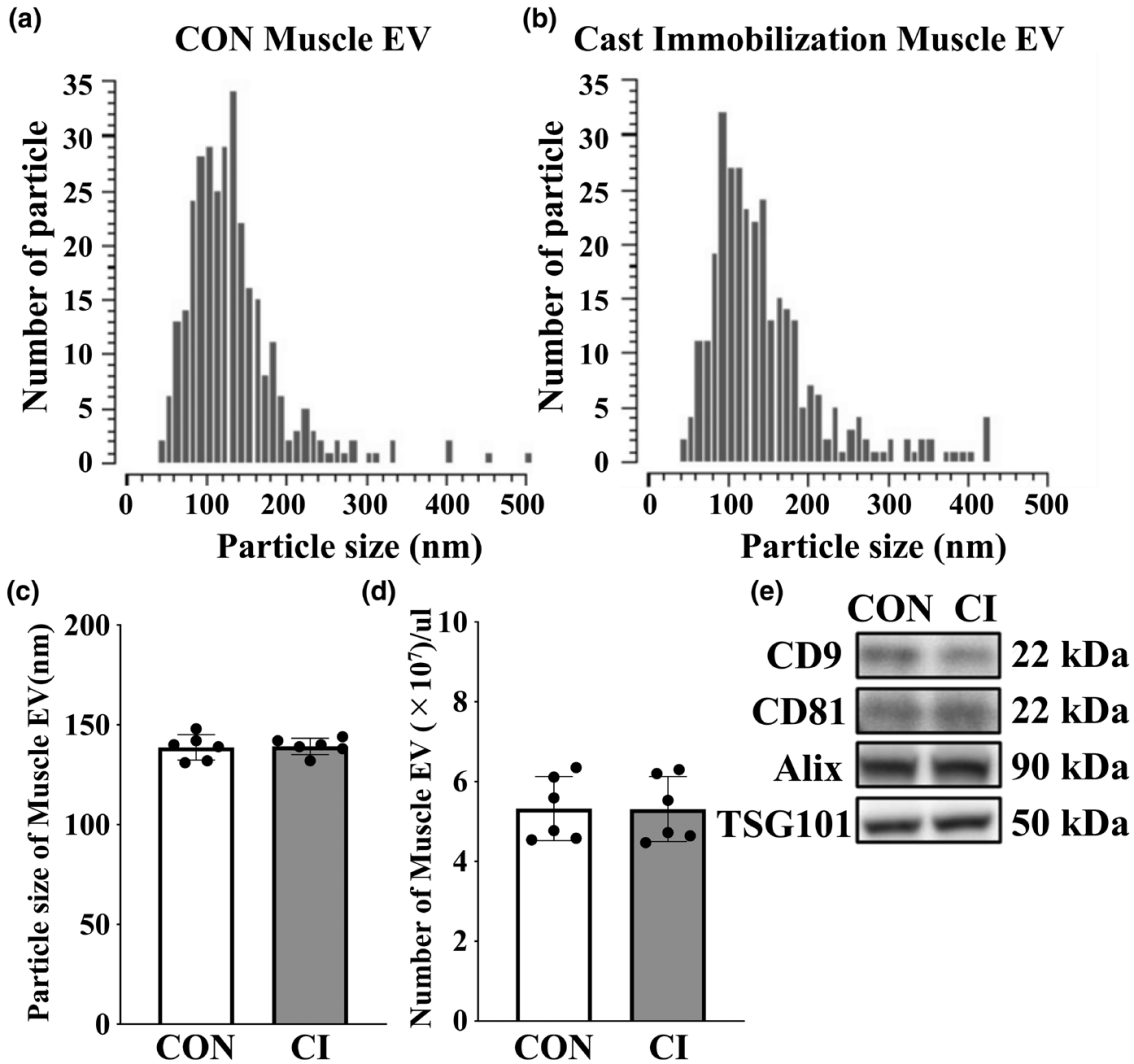
Particle size of extracellular vesicles isolated from the skeletal muscle culture medium of control mice (a) and cast immobilization mice (b) (*n* = 6/each group). (c) Particle size of extracellular vesicles comparison between two groups. (d) Number of extracellular vesicles contained in the culture medium comparison between two groups. (e) CD9, CD81, Alix, and TSG101, an exosome marker, protein expression of extracellular vesicles isolated from the skeletal muscle. Values represent mean ± SD.

The particle sizes of extracellular vesicles isolated from the culture medium of mice skeletal muscle were as follows: control mice, 138.6 ± 4.7 nm (Figure 1a); cast immobilization mice, 139.7 ± 2.8 nm (Figure 1b). There was no significant difference in the particle size of the extracellular vesicles between the two groups (Figure 1c). There was no significant difference in the number of exosomes contained in the culture medium (Figure 1d). Protein expression of CD9, CD81, Alix, and TSG101, an exosome marker, was detected in extracellular vesicles isolated from the culture medium of mice in all groups (Figure 1e).

### 3.3 Cast immobilization‐induced miRNA expression changes in skeletal muscle‐derived extracellular vesicles

**FIGURE 2:**
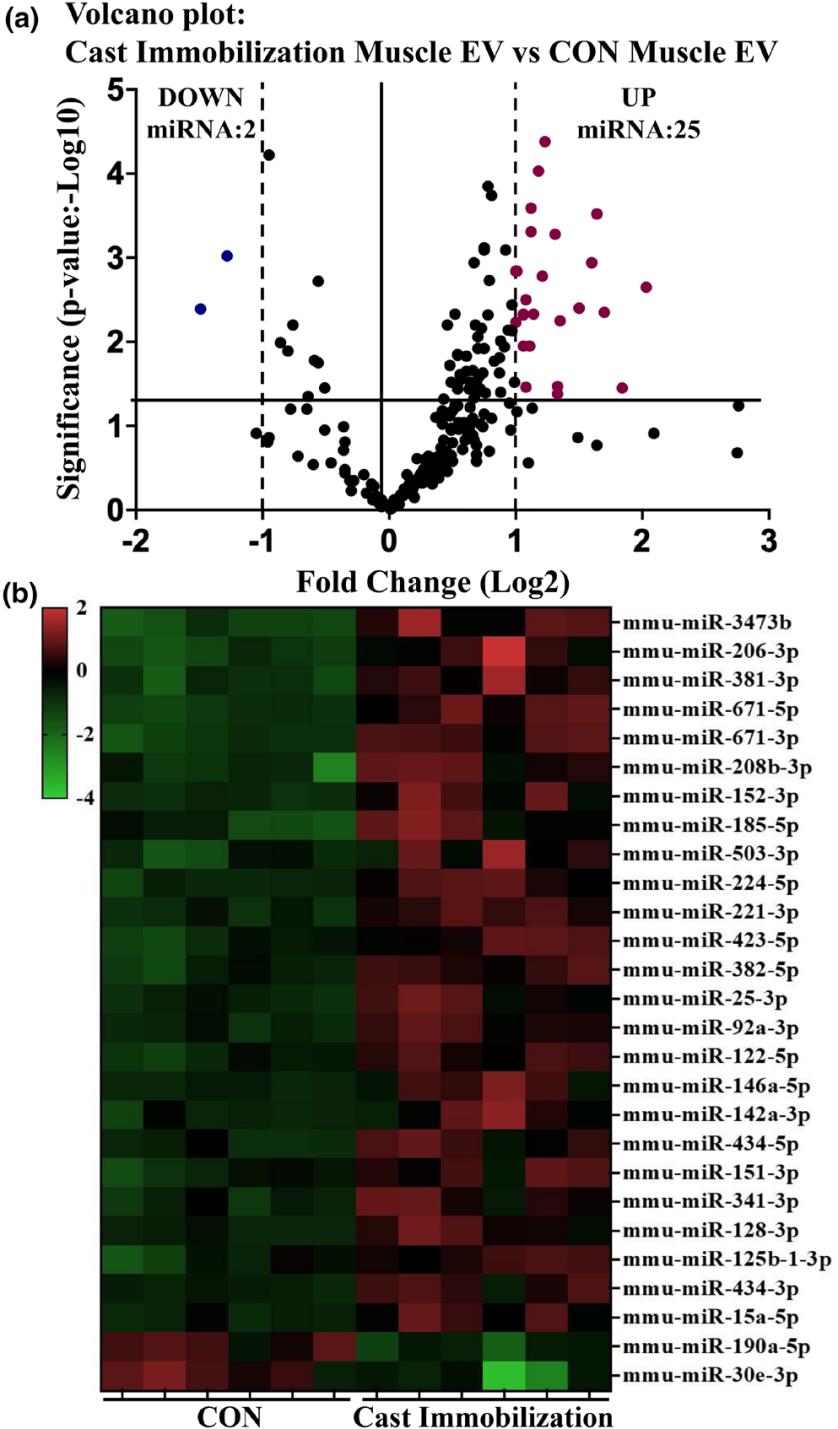
(a) Volcano plot of small RNA‐seq data displaying the pattern of gene expression values for cast immobilization group relative to the control group (*n* = 6/each group). Significant differentially expressed genes (*p* ≤ 0.05) are highlighted in red and blue, with the dotted line representing the boundary for identification of upregulated or downregulated genes. (b) Heat map of significantly differentially expressed microRNA (miRNA) (>2‐fold in cast immobilization group compared to control group).

We selected microRNAs with at least a 2‐fold (log2: >1 or <−1) change in expression between the cast immobilization and control groups of skeletal muscle‐derived extracellular vesicles (Table S1) and analyzed them using a volcano plot. A total of 27 microRNAs were differentially expressed between the two groups (Figure 2a). In the cast immobilization group, 25 of the 27 microRNAs were upregulated and 2 microRNAs were downregulated compared to the control group (Figure 2a). Figure 2b shows a heat map of the differences in microRNA expression levels between the control and cast immobilization groups. Of the 27 differentially expressed microRNAs, the microRNAs that showed a change in expression >3‐fold (log2: >1.585) between the cast immobilization group and the control group were mmu‐miR‐3473b, mmu‐miR‐206‐3p, mmu‐miR‐381‐3p, mmu‐miR‐671‐5p, mmu‐miR‐671‐3p, and mmu‐miR‐208b‐3p (Figure 2b).

### 3.4 mRNA expression changes in brain neuron cells following treatment with skeletal muscle‐derived extracellular vesicles after cast immobilization

**FIGURE 3:**
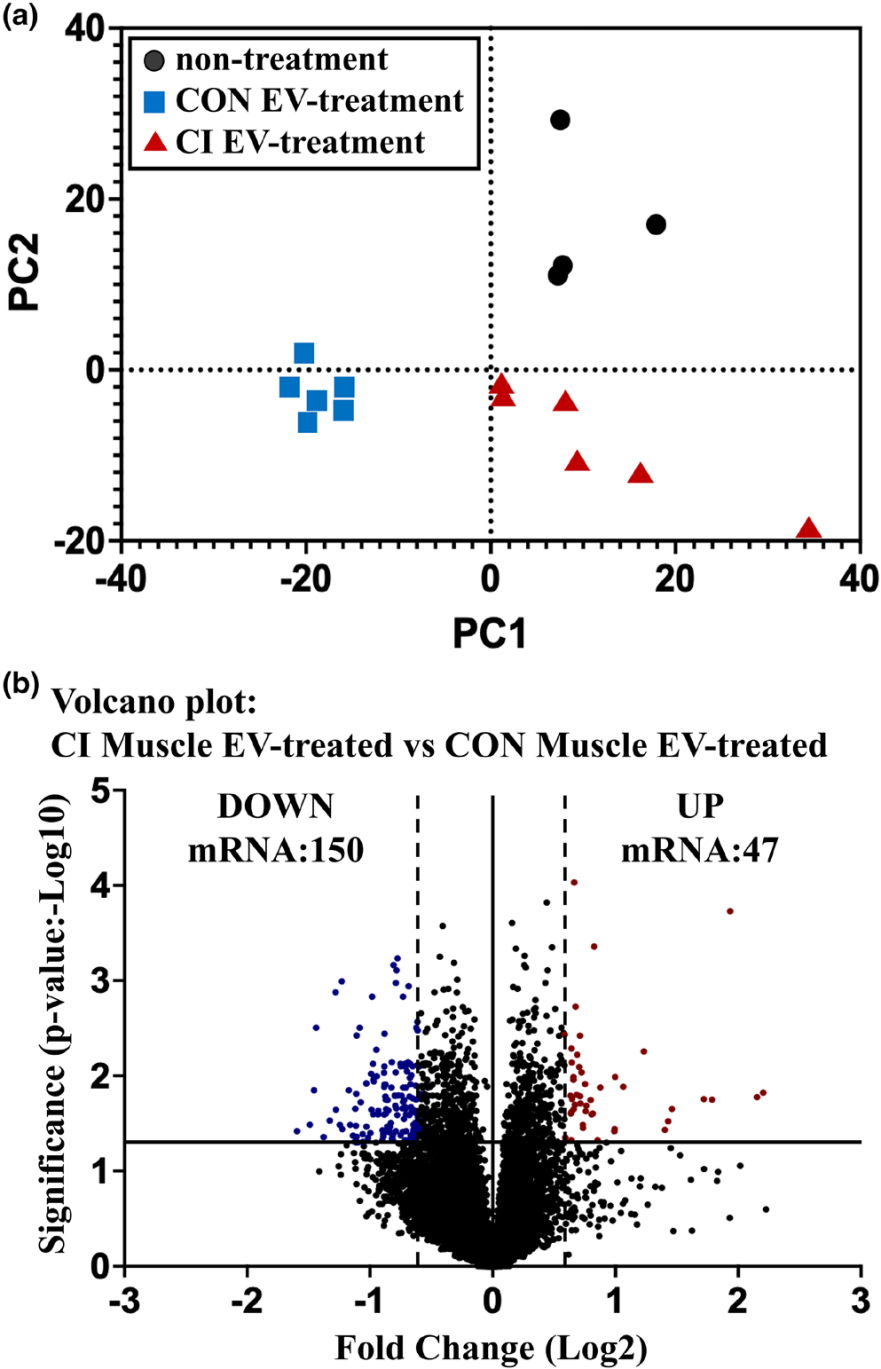
(a) Principal component analysis (PCA) of mRNA expression profiles in brain neuron cells of nontreated (*n* = 4), skeletal muscle‐derived extracellular vesicles from control mice (CON EV)‐ treated (*n* = 6), and skeletal muscle‐derived extracellular vesicles from cast immobilization mice (CI EV)‐ treated (*n* = 6) groups. (b) Volcano plots of mRNA‐seq transcriptome data displaying the pattern of gene expression values for CI EV‐ treatment relative to the CON EV‐ treatment (*n* = 6/each group).

We next performed mRNA‐seq analysis of neuronal cells differentiated from hippocampal neural stem cells that had been treated with skeletal muscle‐derived extracellular vesicles from control mice (CON EV‐treatment), or cast‐immobilized mice (CI EV‐treatment), compared to an untreated control. Principal component analysis (PCA) revealed a tight clustering of samples within each group and a distinct separation in mRNA expression profiles between the groups (Figure 3a). We selected mRNAs showing at least a 1.5‐fold (log2: >0.585 or <−0.585) change in expression between the CI EV‐treatment and CON EV‐treatment groups and analyzed them using a volcano plot. A total of 197 mRNAs were differentially expressed between the CI and CON EV‐treated groups (Figure 3b). Compared to the CON EV treatment group, 47 mRNAs were upregulated and 150 were downregulated in the CI EV treatment group (Figure 3b). The list of differentially expressed mRNAs is provided in Table S2.

Additional comparisons including nontreated brain neuron cells were performed to evaluate the baseline transcriptional effects associated with EV exposure itself. Both CON EV‐treated and CI EV‐treated neurons showed transcriptional alterations relative to untreated controls (Figure S1).

**FIGURE 4:**
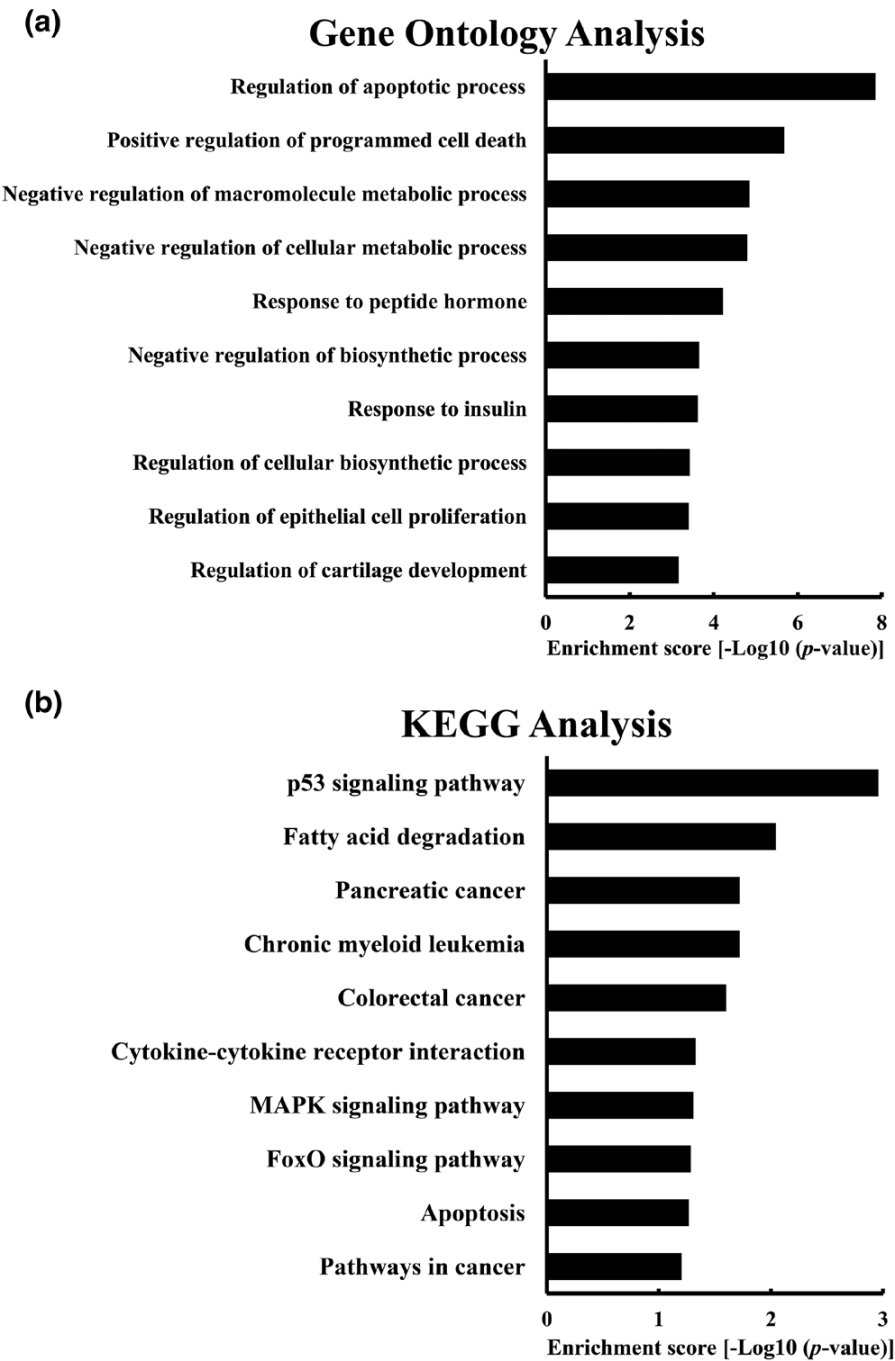
GO enrichment and KEGG pathway analysis of mRNAs increased in brain neuron cells by treatment with extracellular vesicles derived from atrophic skeletal muscle. (a) Top 10 functional GO terms for differentially expressed mRNA. (b) Top 10 signaling pathways predicted to be regulated by the differentially expressed mRNA.

We analyzed the involvement of the 47 mRNAs up‐regulated in the CI EV‐treated groups in biological processes and signaling pathways using GO enrichment analysis and KEGG pathway analysis. A list of GO terms and KEGG signaling pathways with an enrichment score of *p* < 0.10 is shown in Table S3. GO analysis showed that CI EV‐treatment significantly affected the regulation of apoptotic processes and negatively regulated cellular metabolic processes (Figure 4a). KEGG analysis showed that CI‐EV treatment activated the p53, MAPK, FoxO signaling pathway and apoptosis (Figure 4b).

## 4 DISCUSSION

The secretion of extracellular vesicles triggered by skeletal muscle atrophy may play an important role in sarcopenia‐related cognitive impairment. However, the effects of muscle atrophy on the properties of skeletal muscle‐derived extracellular vesicles have not been extensively investigated. In this study, we investigated the effects of cast immobilization‐induced muscle atrophy on the microRNA profiles of skeletal muscle‐derived extracellular vesicles. We found that the expression levels of 27 microRNAs were altered in muscle‐derived extracellular vesicles 14 days after cast immobilization. Notably, the expression levels of miR‐3473b, miR‐206, miR‐381, and miR‐671 increased >3‐fold. Previous studies have reported that some of these microRNAs are highly expressed in extracellular vesicles secreted into the culture medium of starvation‐induced atrophic myotubes (Shao et al., 2022). These results indicate that muscle atrophy promotes the secretion of extracellular vesicles enriched with specific microRNAs. MicroRNAs that are strongly expressed in atrophic skeletal muscle‐derived extracellular vesicles may play an important role in the pathogenesis of muscle atrophy‐related cognitive impairment caused by aging and inactivity. Previous studies have revealed that some miRNAs enriched in extracellular vesicles secreted from atrophic skeletal muscles play a role in regulating brain function. It has been reported that the expression level of miR‐206 is increased in the brains of patients with Alzheimer's disease and in model mice, and that the administration of an miR‐206 inhibitor improves memory function via neurogenesis in Alzheimer's disease model mice (Lee et al., 2012). We found that the microRNAs miR‐125b, miR‐128, miR‐142a, and miR‐146a are enriched in extracellular vesicles secreted from atrophic skeletal muscle, and these microRNAs have been suggested to be involved in the development of Alzheimer's disease (Song & Kim, 2017; Jin et al., 2018; Lanza et al., 2023; Zhan‐Qiang et al., 2023). For example, Zhan‐Qiang et al. reported that the expression level of miR‐146a in the brain increases in an Alzheimer's disease mouse model, and that the administration of a miR‐146a inhibitor improves memory function in Alzheimer disease model mice (Zhan‐Qiang et al., 2023). This suggests that extracellular vesicles secreted from skeletal muscles atrophied by immobilization are rich in miRNAs related to the development of Alzheimer's disease and may therefore affect brain neurons via endocrine mechanisms.

To investigate the effect of atrophic skeletal muscle‐derived extracellular vesicles secreted by cast immobilization‐induced inactivity on cell function in brain neurons, we examined comprehensive changes in mRNA expression following treatment of neurons with skeletal muscle‐derived extracellular vesicles. This revealed that treatment with atrophic skeletal muscle‐derived extracellular vesicles dramatically altered the mRNA expression profiles of brain neurons. Although several miRNAs exhibited relatively modest log2 fold changes, microRNAs are known to exert biologically relevant regulatory effects even with relatively small expression alterations. In contrast, interpretation of neuronal transcriptional responses may require greater caution, particularly for genes showing modest fold changes. We next performed microRNA‐mRNA target analysis using TarBase v9.0. Table S4 shows a list of predicted microRNAs targeting mRNAs altered by CI EV treatment. We found that microRNAs that were altered in atrophic skeletal muscle‐derived EVs in mice by cast immobilization were abundantly contained in the targets of mRNAs that were altered in brain neuron cells. We also evaluated the molecular function of 47 mRNAs that were upregulated in brain neurons by GO analysis and found that they were enriched in key genes that positively regulate programmed cell death, such as apoptosis. Furthermore, KEGG analysis indicated that atrophic skeletal muscle‐derived extracellular vesicles activate various signaling pathways in brain neurons. Importantly, these signaling pathways include the p53 and MAPK signaling pathways, which regulate apoptosis induction. Although apoptosis‐ and FoxO‐related pathways did not meet the predefined threshold for statistical significance (*p* < 0.05), both pathways showed trend‐level enrichment (*p* < 0.10). In addition, several apoptosis‐associated genes exhibited contrasting expression patterns between CON EV‐ and CI EV‐treated neurons. Therefore, these findings may suggest potential modulation of neuronal stress‐response pathways by atrophic muscle‐derived EVs, although further validation will be required. Previous studies have shown that the activation of the p53 and MAPK signaling pathways is increased in the brains of patients with Alzheimer's disease and elderly subjects, and that the activation of these pathways is related to the progression of Alzheimer's disease by inducing apoptosis, oxidative stress, and chronic inflammation in brain neuronal cells (Chakraborty et al., 2023; Wolfrum et al., 2022). Importantly, it has been shown that miRNA‐125b and miR‐128, which are enriched in extracellular vesicles secreted from atrophic skeletal muscle, induce apoptosis of neuron cells (Jin et al., 2018; Lanza et al., 2023). Therefore, it is possible that the microRNAs that increase in extracellular vesicles secreted from atrophic skeletal muscle synergistically cause brain dysfunction via apoptosis of brain neurons.

Interestingly, several apoptosis‐related genes (*Lifr, Egr3*, *Gadd45b*, *Phlda3*, *Srsf6*, *Stk40*, *Snai2*, *Tnfrsf10b*, *Zfas1*) tended to be downregulated in neurons treated with CON EVs relative to untreated controls, whereas CI EV treatment showed an opposite tendency toward increased or no change expression of apoptosis‐related genes. These findings suggest that muscle‐derived EVs may contribute to maintenance of neuronal homeostasis or suppression of stress‐related signaling, while EVs derived from atrophic muscle may alter this regulatory effect and promote transcriptional programs associated with neuronal stress or apoptosis. However, because the number of nontreated neuronal samples was smaller than that of the EV‐treated groups, comparisons involving untreated controls may have had reduced statistical power. Therefore, these observations should be interpreted cautiously and require further functional validation in future studies.

Although the present transcriptomic analyses suggest that muscle‐derived EVs, particularly those derived from atrophic muscle, may influence apoptosis‐related pathways in brain neurons, several limitations should be considered. First, the current study primarily relies on RNA‐seq–based observations, and direct functional validation of neuronal phenotypes was not performed. Therefore, it remains unclear whether the observed transcriptional alterations translate into measurable changes in neuronal viability, apoptosis, proliferation, differentiation, or metabolic function. Future studies incorporating functional assays, such as cell viability measurements, apoptosis assays, neurite morphology analysis, or metabolic profiling, will be important to further clarify the biological significance of these EV‐mediated effects. Such approaches may help determine whether the molecular alterations observed in the present study contribute to functional neuronal dysfunction under atrophy‐related conditions.

Several limitations associated with the explant‐based EV collection method should be considered when interpreting the present findings. Because EVs were collected following tissue excision and ex vivo culture, it is possible that tissue handling, altered oxygen tension, nutrient availability, or other stress‐related factors influenced EV release and cargo composition. Therefore, the isolated EVs may not fully reflect the physiological in vivo extracellular vesicle population present under native conditions. Such ex vivo influences could potentially contribute to the observed differences in miRNA profiles and downstream neuronal transcriptional responses, including apoptosis‐related pathways. Future studies incorporating in vivo EV isolation strategies, functional validation, and additional EV characterization approaches will be important to further establish the physiological relevance of muscle‐derived EV signaling under atrophy‐related conditions.

In this study, we found that muscle atrophy induced by cast immobilization altered the microRNA profiles in skeletal muscle‐derived extracellular vesicles, as well as the mRNA profiles in brain neurons exposed to these vesicles. We present evidence that changes in the properties of skeletal muscle‐derived extracellular vesicles induced by disuse‐induced muscle atrophy may play an important role in the development of neurodegenerative diseases via neuronal apoptosis. Nevertheless, the precise biological functions of atrophy‐induced skeletal muscle‐derived extracellular vesicles remain unclear. Future studies are warranted to investigate the effects of administering atrophic skeletal muscle‐derived extracellular vesicles on cognitive function in mice in vivo.

## AUTHOR CONTRIBUTIONS

**Noriaki Kawanishi:** Conceptualization; data curation; formal analysis; funding acquisition; investigation; methodology; project administration; resources; software; supervision; validation; visualization. **Shuichi Machida:** Formal analysis; methodology; project administration. **Katsuhiko Suzuki:** Methodology; supervision.

## FUNDING INFORMATION

This work was supported by a grant‐in‐aid for the Japan Society for the Promotion of Science KAKENHI Grant No. 23H03274 (N. Kawanishi) and 20H00574 (K. Suzuki).

## CONFLICT OF INTEREST STATEMENT

The authors declare no conflicts of interest.

## ETHICS STATEMENT

Experimental procedures were approved by the Institutional Animal Care and Use Committee of the China Institute of Technology (approval number: 20230101), in accordance with the Guiding Principles for the Care and Use of Animals. Animal handling and reporting throughout the study were adhered to the ARRIVE (Animal Research: Reporting of In Vivo Experiments) guidelines.

## Supporting information


**Figure S1.** Volcano plots of mRNA‐seq transcriptome data displaying the pattern of gene expression values for CON EV‐ treatment relative to the nontreatment.
**Figure S2.** (A) GO enrichment analysis of mRNA decreased in cell CON EV‐ treatment compared to nontreatment (Top 20 functional GO terms). (B) GO enrichment analysis of mRNA increased CON EV‐ treatment compared to nontreatment (Top 20 functional GO terms).
**Figure S3.** Volcano plots of mRNA‐seq transcriptome data displaying the pattern of gene expression values for CI EV‐ treatment relative to the nontreatment.
**Figure S4.** (A) GO enrichment analysis of mRNA decreased in cell CI EV‐ treatment compared to nontreatment (Top 20 functional GO terms). (B) GO enrichment analysis of mRNA increased in CI EV‐ treatment compared to nontreatment (Top 20 functional GO terms).

**Table S1.** List of microRNAs that show a significant change in expression (>2‐fold) in the cast immobilization group compared to that in the control.

**Table S2.** List of mRNAs that show a significant change in expression (>1.5 fold) in the CI EV‐treated brain neuron cells compared to that in the CON EV‐treated brain neuron cells. CI, cast immobilization; CON, control.

**Table S3.** List of GO terms and KEGG signaling pathways with an enrichment score of p1.

**Table S4.** List of predicted miRNAs that targets mRNAs that show a significant change in expression (>1.5 fold) in the CI EV‐treated brain neuron cells compared to that in the CON EV‐treated brain neuron cells.

## Data Availability

Sequencing data have been deposited in the DNA Data Bank of Japan Sequence Read Archive (accession number: DRA020350).
