# Supplementary material for: Extracellular vesicles derived from immobilization‐induced atrophic muscle of male mice contain distinct microRNA profiles and impact mRNA profiles in brain neurons
Source: Physiol Rep. 2026 Aug 13;14(16):e71052. doi: 10.14814/phy2.71052 (PMC13473741; doi:10.14814/phy2.71052)
Supplement: Supplementary file 1 [file PHY2-14-e71052-s001.pdf]

Supplementary Table S1. List of microRNAs that show a significant change in expression (>2-fold) in the cast immobilization group compared to that in the control

Cast immobilization groups vs Control groups					
Up-Regulation			Down-Regulation		
miRNA species	change (log2)	p value	miRNA species	change (log2)	p value
mmu-miR-3473b	2.03	0.002	mmu-miR-190a-5p	-1.28	0.001
mmu-miR-206-3p	1.84	0.035	mmu-miR-30e-3p	-1.49	0.004
mmu-miR-381-3p	1.70	0.004			
mmu-miR-671-5p	1.64	0.000			
mmu-miR-671-3p	1.60	0.001			
mmu-miR-208b-3p	1.50	0.004			
mmu-miR-671-5p	1.64	0.000			
mmu-miR-671-3p	1.60	0.001			
mmu-miR-208b-3p	1.50	0.004			
mmu-miR-152-3p	1.35	0.006			
mmu-miR-185-5p	1.33	0.034			
mmu-miR-503-3p	1.33	0.041			
mmu-miR-224-5p	1.31	0.001			
mmu-miR-221-3p	1.23	0.000			
mmu-miR-423-5p	1.21	0.002			
mmu-miR-382-5p	1.18	0.000			
mmu-miR-25-3p	1.14	0.005			
mmu-miR-92a-3p	1.12	0.000			
mmu-miR-122-5p	1.12	0.000			
mmu-miR-146a-5p	1.11	0.011			
mmu-miR-142a-3p	1.08	0.035			
mmu-miR-434-5p	1.08	0.003			
mmu-miR-151-3p	1.06	0.005			
mmu-miR-341-3p	1.06	0.011			
mmu-miR-128-3p	1.06	0.005			
mmu-miR-125b-1-3p	1.01	0.001			
mmu-miR-434-3p	1.00	0.001			
mmu-miR-15a-5p	1.00	0.006			
