# Supplementary material for: Extracellular vesicles derived from immobilization‐induced atrophic muscle of male mice contain distinct microRNA profiles and impact mRNA profiles in brain neurons
Source: Physiol Rep. 2026 Aug 13;14(16):e71052. doi: 10.14814/phy2.71052 (PMC13473741; doi:10.14814/phy2.71052)
Supplement: Supplementary file 2 [file PHY2-14-e71052-s002.pdf]

Supplementary Table S2. List of mRNAs that show a significant change in expression (>1.5 fold) in the CI EV-treated brain neuron cells compared to that in the CON EV-treated brain neuron cells. CI, cast immobilization; CON, control.

CI EV-treated brain neuron cells vs CON EV-treated brain neuron cells.					
Up-Regulation			Down-Regulation		
mRNA species	change (log2)	p value	mRNA species	Fold change (log2)	p value
Gadd45b	2.20	0.015	Gm20356	-1.60	0.038
Srsf6	2.15	0.017	Olfr723	-1.49	0.033
Lifr	1.93	0.000	Arhgap33os	-1.46	0.014
Eif2ak2	1.79	0.018	0610010F05Rik	-1.44	0.003
Acsbg1	1.72	0.018	Olfr825	-1.38	0.044
Ntf3	1.46	0.022	Gigyf1	-1.33	0.029
Tnfrsf10b	1.43	0.030	3300002A11Rik	-1.28	0.001
Acadl	1.40	0.037	Tm4sf5	-1.28	0.023
Egr1	1.23	0.006	Spata19	-1.25	0.033
Phlda3	1.06	0.013	Mtrf1	-1.23	0.001
Slc2a1	1.00	0.010	4932415M13Rik	-1.22	0.036
Gdpd2	0.99	0.036	AY761185	-1.17	0.014
Snai2	0.99	0.035	Gm30818	-1.16	0.033
Htra1	0.88	0.013	Tmem190	-1.14	0.042
Rhbdl3	0.85	0.047	Olfr1469	-1.12	0.049
Atf3	0.83	0.000	Dcst1	-1.12	0.022
Kifc1	0.81	0.025	Gm4285	-1.12	0.033
Pkp2	0.81	0.025	Irf2bp1	-1.11	0.015
Gadd45g	0.80	0.018	Pcsk4	-1.11	0.004
Cers1	0.76	0.021	Kng2	-1.11	0.043
Tgfbr1	0.76	0.023	Leap2	-1.10	0.050
Natd1	0.75	0.012	Cd2	-1.08	0.003
Eci1	0.74	0.035	1700020D05Rik	-1.08	0.019
Impa2	0.73	0.033	Olfr1014	-1.06	0.041
Zfas1	0.72	0.009	LOC108168109	-1.06	0.049
Rnf169	0.71	0.019	1700036A12Rik	-1.03	0.012
Prkx	0.71	0.016	Pcdhb19	-1.03	0.012
Sertad1	0.71	0.004	Zcchc8	-1.03	0.034
Med26	0.70	0.008	Gnat1	-1.02	0.045
Fblim1	0.69	0.006	LOC102641859	-1.00	0.040
Ubap1l	0.68	0.016	Olfr91	-0.99	0.010
Egr3	0.68	0.002	Nek4	-0.99	0.023
Trib3	0.67	0.020	Abcc8	-0.98	0.001
Sesn2	0.67	0.000	Lama2	-0.98	0.007
Zfp383	0.66	0.022	Olfr248	-0.97	0.025
Slc19a2	0.66	0.011	Tdgf1	-0.97	0.011
Pfdn5	0.66	0.010	L3hypdh	-0.97	0.011
Stk40	0.65	0.017	Dnase1l2	-0.97	0.010
Btg2	0.64	0.007	Itgal	-0.95	0.005
Plod1	0.64	0.047	Gm3230	-0.94	0.010

Bmpr1b	0.64	0.005
Bach1	0.64	0.024
Errfi1	0.64	0.025
Spon1	0.63	0.016
Rara	0.59	0.045
Chac1	0.59	0.048
Egr4	0.59	0.004

Abhd16b	-0.93	0.025
4930596I21Rik	-0.93	0.023
Gm30896	-0.90	0.047
Gm33349	-0.90	0.037
Rftn1	-0.89	0.043
Krtap4-9	-0.89	0.041
Msln	-0.88	0.009
Zfp513	-0.88	0.025
Gpbar1	-0.88	0.013
Cldn5	-0.88	0.008
Fam46a	-0.88	0.004
Npbwr1	-0.88	0.012
Kif22	-0.87	0.039
Cdyl	-0.87	0.012
Olfr657	-0.87	0.017
Olfr1245	-0.87	0.025
Klf16	-0.87	0.018
Taar7d	-0.87	0.022
Olfr1039	-0.86	0.034
Olfr875	-0.86	0.018
Tas2r138	-0.85	0.023
Olfr1204	-0.85	0.034
LOC102640328	-0.84	0.039
Gm2164	-0.84	0.039
Rel	-0.84	0.034
Olfr1494	-0.83	0.031
LOC108168936	-0.82	0.013
Pqlc3	-0.82	0.007
Odf3b	-0.82	0.045
Gm5766	-0.81	0.042
Krt4	-0.81	0.047
Diaph2	-0.81	0.001
Gm5382	-0.80	0.016
Zc3h6	-0.79	0.016
Tmem62	-0.79	0.021
Olfr1231	-0.79	0.038
Ubfd1	-0.79	0.009
Reep6	-0.79	0.001
LOC108167746	-0.79	0.022
LOC108167536	-0.78	0.001
Hbb-bh1	-0.78	0.026
Slc22a8	-0.78	0.001
Olfr1406	-0.77	0.016
D630041G03Rik	-0.77	0.016
Ifne	-0.75	0.007
Olfr223	-0.75	0.042
LOC108168808	-0.75	0.008
Olfr1339	-0.74	0.016
Olfr54	-0.73	0.022
Olfr1428	-0.73	0.039

Taar5	-0.73	0.001
Tmem135	-0.73	0.019
2210408F21Rik	-0.73	0.013
9130409J20Rik	-0.72	0.028
Gm12500	-0.72	0.018
Ptger4	-0.72	0.047
Lrrc3b	-0.71	0.007
Setdb2	-0.71	0.021
Gm12633	-0.71	0.022
Gm11555	-0.71	0.008
Ercc4	-0.71	0.025
Lig1	-0.71	0.013
Pcgf5	-0.71	0.038
D630024D03Rik	-0.70	0.047
H60b	-0.70	0.009
5730409E04Rik	-0.69	0.007
LOC108168307	-0.68	0.001
Poc1b	-0.68	0.026
Dock4	-0.68	0.017
Dock5	-0.68	0.010
Wdr4	-0.67	0.007
Macf1	-0.67	0.012
Tmem200b	-0.67	0.042
Chst14	-0.67	0.021
Tfdp2	-0.67	0.042
Stx1a	-0.66	0.043
A330076H08Rik	-0.66	0.010
Ptchd1	-0.66	0.024
Chrm5	-0.65	0.008
Cdc25a	-0.65	0.017
Mpdz	-0.65	0.023
Sass6	-0.65	0.038
Zfp52	-0.65	0.033
Thnsl2	-0.64	0.039
Iqcc	-0.64	0.049
LOC108169077	-0.64	0.036
LOC108167889	-0.64	0.039
Wdr86	-0.64	0.024
Slc23a2	-0.63	0.031
LOC108168108	-0.63	0.024
1700066C05Rik	-0.63	0.044
Tdpoz2	-0.63	0.041
Olfr419	-0.63	0.013
2210420H20Rik	-0.62	0.018
Pcdhb8	-0.62	0.003
Recql4	-0.62	0.035
Bsn	-0.62	0.040
Fam171a1	-0.61	0.008
Pacsin1	-0.61	0.003
Rimbp3	-0.61	0.003

**1110008L16Rik**	**-0.61**	**0.025**
**Gm3837**	**-0.61**	**0.027**
**Alox12e**	**-0.61**	**0.034**
**Ypel1**	**-0.61**	**0.029**
**Nppc**	**-0.60**	**0.008**
**Ryr2**	**-0.59**	**0.025**
**Olfr1505**	**-0.59**	**0.036**
**Rnf7**	**-0.59**	**0.015**
**Tmem196**	**-0.59**	**0.038**
**Zfp444**	**-0.59**	**0.024**
