# Supplementary material for: Extracellular vesicles derived from immobilization‐induced atrophic muscle of male mice contain distinct microRNA profiles and impact mRNA profiles in brain neurons
Source: Physiol Rep. 2026 Aug 13;14(16):e71052. doi: 10.14814/phy2.71052 (PMC13473741; doi:10.14814/phy2.71052)
Supplement: Supplementary file 3 [file PHY2-14-e71052-s003.pdf]

Supplementary Table S3. List of GO terms and KEGG signaling pathways with an enrichment score of p<0.10 (-log10: >1).

GO (Gene Ontology) analysis		
GO term	Genes Count	P-Value (-log10)
regulation of apoptotic process	19	7.854
positive regulation of programmed cell death	11	5.678
negative regulation of macromolecule metabolic process	20	4.854
negative regulation of cellular metabolic process	19	4.796
response to peptide hormone	8	4.222
negative regulation of biosynthetic process	16	3.658
response to insulin	6	3.620
regulation of cellular biosynthetic process	25	3.432
regulation of epithelial cell proliferation	7	3.409
regulation of cartilage development	4	3.167
negative regulation of nucleobase-containing compound metabolic process	12	3.155
regulation of macromolecule biosynthetic process	24	3.102
protein phosphorylation	8	3.097
endothelial cell migration	4	3.076
regulation of nucleobase-containing compound metabolic process	20	2.959
regulation of signal transduction	17	2.921
negative regulation of cell population proliferation	8	2.854
negative regulation of transferase activity	5	2.699
regulation of protein metabolic process	14	2.699
epithelial cell migration	4	2.638
negative regulation of cartilage development	3	2.602
regulation of RNA metabolic process	18	2.481
phosphorylation	8	2.469
negative regulation of signal transduction	10	2.456
negative regulation of programmed cell death	9	2.432
positive regulation of cell population proliferation	9	2.432
intracellular signal transduction	11	2.409
positive regulation of nucleobase-containing compound metabolic process	12	2.377
intrinsic apoptotic signaling pathway in response to endoplasmic reticulum stress	3	2.367
regulation of intracellular signal transduction	12	2.310
negative regulation of cell communication	10	2.194
cellular response to amino acid starvation	3	2.161

regulation of chondrocyte differentiation	3	2.143
regulation of epithelial cell differentiation	4	2.125
intrinsic apoptotic signaling pathway	4	2.119
positive regulation of macromolecule metabolic process	16	2.027
circulatory system development	8	2.009
cartilage development	4	2.000
regulation of kinase activity	6	1.959
negative regulation of cell differentiation	7	1.959
positive regulation of cellular metabolic process	15	1.886
enzyme-linked receptor protein signaling pathway	6	1.854
regulation of lipid biosynthetic process	4	1.796
regulation of apoptotic signaling pathway	5	1.770
positive regulation of biosynthetic process	13	1.745
fatty acid oxidation	3	1.745
fatty acid catabolic process	3	1.699
lipid oxidation	3	1.678
regulation of stem cell proliferation	3	1.620
regulation of translation in response to endoplasmic reticulum stress	2	1.585
positive regulation of cell differentiation	7	1.569
regulation of chemokine production	3	1.553
negative regulation of lipid metabolic process	3	1.481
skeletal system development	5	1.469
negative regulation of small molecule metabolic process	3	1.432
apoptotic signaling pathway	4	1.398
kidney development	4	1.301
regulation of osteoblast differentiation	3	1.284
reproductive structure development	4	1.268
renal system development	4	1.268
vasculature development	5	1.260
reproductive system development	4	1.252
regulation of lipid metabolic process	4	1.244
regulation of cellular ketone metabolic process	3	1.237
cardiac epithelial to mesenchymal transition	2	1.229
cellular response to radiation	3	1.222
fatty acid metabolic process	4	1.222
skeletal muscle tissue development	3	1.208
lipid modification	3	1.194
nervous system development	10	1.174
skeletal muscle organ development	3	1.143

cell surface receptor signaling pathway	9	1.108
cellular lipid catabolic process	3	1.092
hexose transmembrane transport	2	1.092
regulation of cell migration	6	1.076
eye development	4	1.071
cell surface receptor protein serine/threonine kinase signaling pathway	3	1.066
visual system development	4	1.066
sensory system development	4	1.051
pallium development	3	1.041
regulation of hematopoietic progenitor cell differentiation	2	1.036
central nervous system development	6	1.032
cardiac muscle tissue development	3	1.032
positive regulation of signal transduction	8	1.027
striated muscle tissue development	3	1.013
carboxylic acid catabolic process	3	1.013
cellular response to glucose starvation	2	1.009
protein localization to plasma membrane	3	1.004

KEGG (Kyoto Encyclopedia of Genes and Genomes) analysis		
KEGG term	Genes Count	P-Value (-log10)
p53 signaling pathway	4	2.95861
Fatty acid degradation	3	2.04576
Pancreatic cancer	3	1.72125
Chronic myeloid leukemia	3	1.72125
Colorectal cancer	3	1.60206
Cytokine-cytokine receptor interaction	4	1.3279
MAPK signaling pathway	4	1.3098
FoxO signaling pathway	3	1.284
Apoptosis	3	1.26761
Pathways in cancer	5	1.20066
Gastric cancer	3	1.18709
Hippo signaling pathway	3	1.14874
Hepatocellular carcinoma	3	1.07572
Cellular senescence	3	1.04576
Thyroid cancer	2	1.00877
