# Supplementary material for: Extracellular vesicles derived from immobilization‐induced atrophic muscle of male mice contain distinct microRNA profiles and impact mRNA profiles in brain neurons
Source: Physiol Rep. 2026 Aug 13;14(16):e71052. doi: 10.14814/phy2.71052 (PMC13473741; doi:10.14814/phy2.71052)
Supplement: Supplementary file 4 [file PHY2-14-e71052-s004.pdf]

Supplementary Figure S1

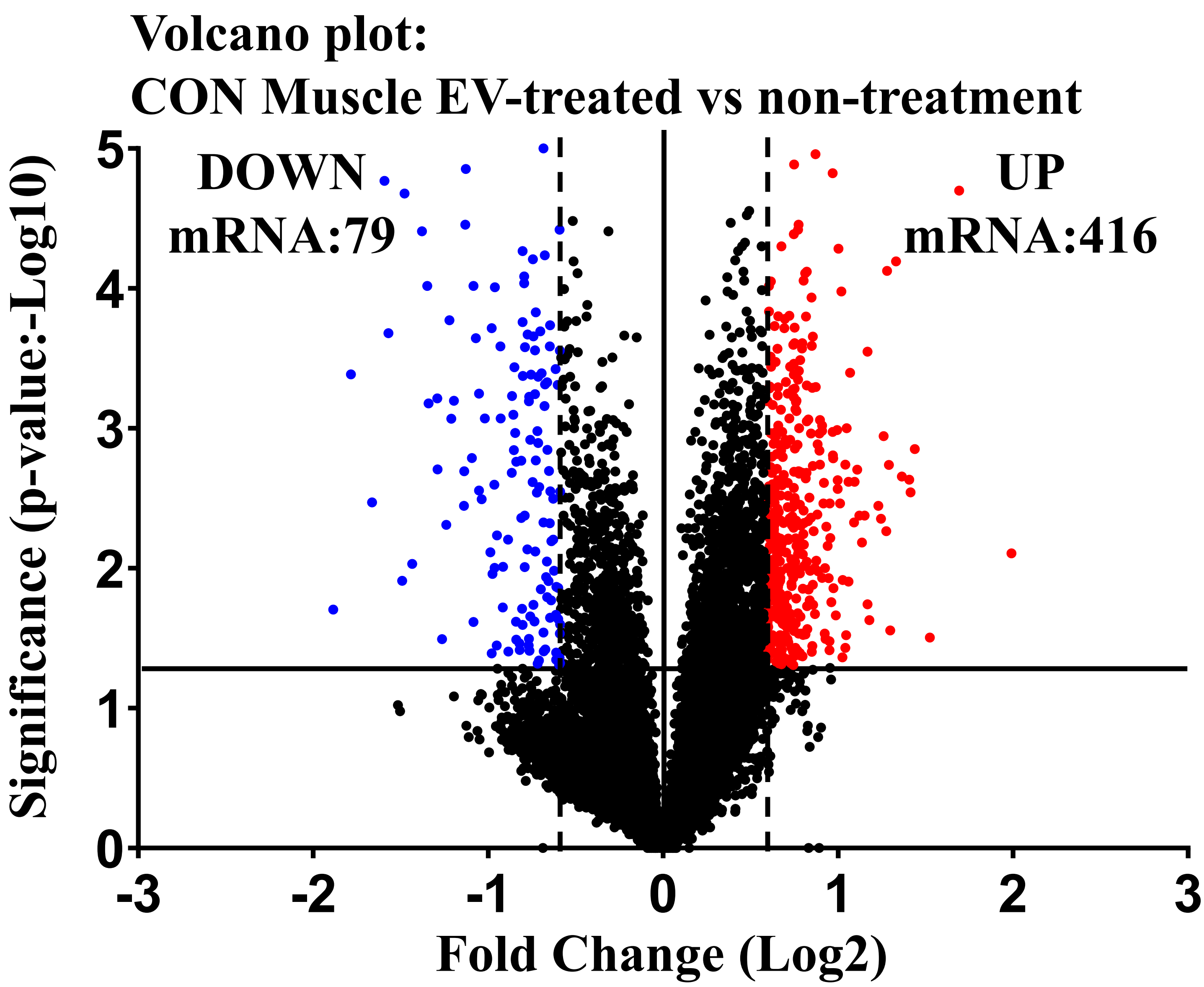

Volcano plots of mRNA-seq transcriptome data displaying the pattern of gene expression values for CON EV- treatment relative to the non- treatment.

Supplementary Figure S2

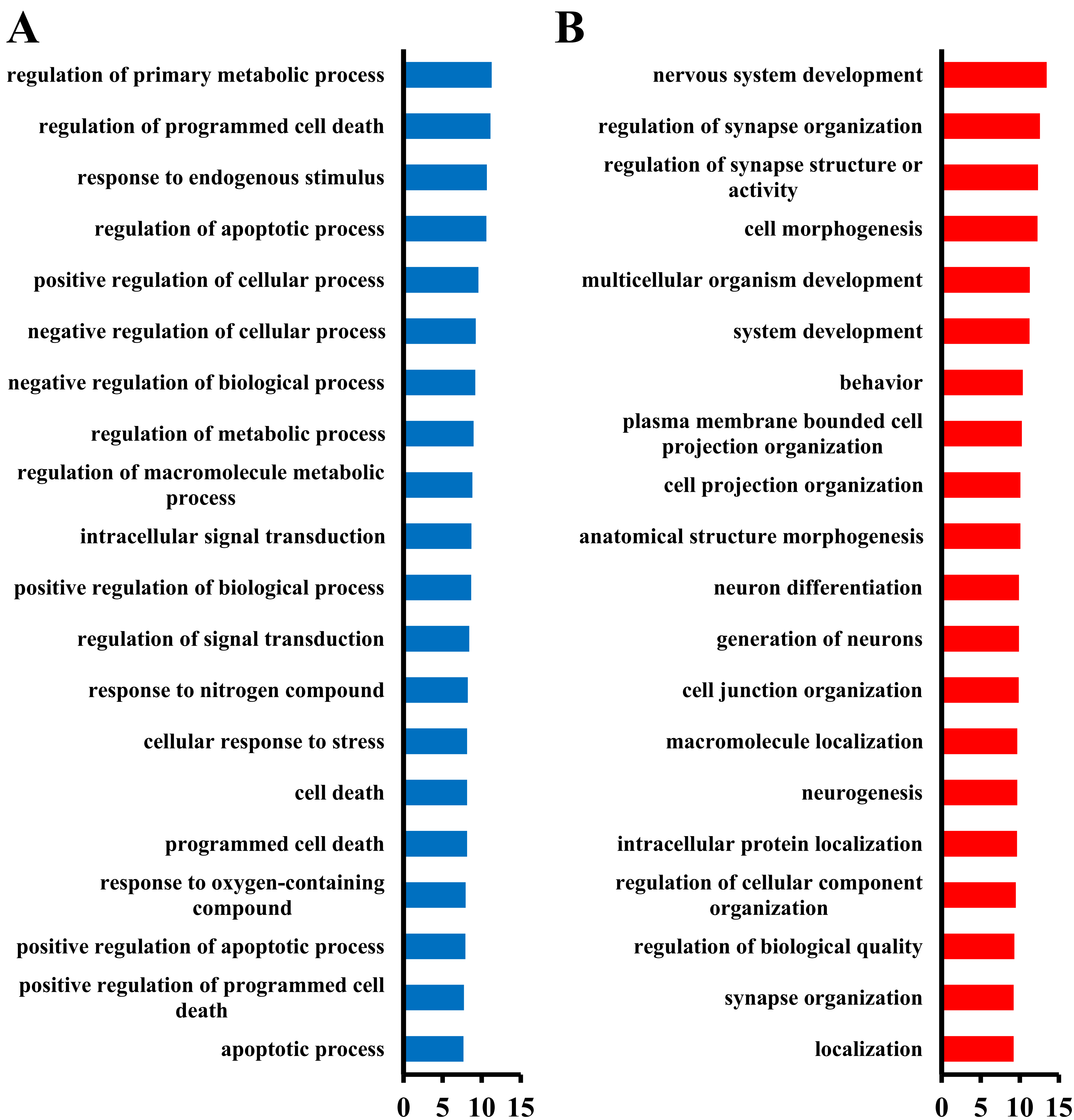

(A) GO enrichment analysis of mRNA decreased in cell CON EV- treatment compared to non- treatment (Top 20 functional GO terms). (B) GO enrichment analysis of mRNA increased CON EV- treatment compared to non- treatment (Top 20 functional GO terms).

Supplementary Figure S3

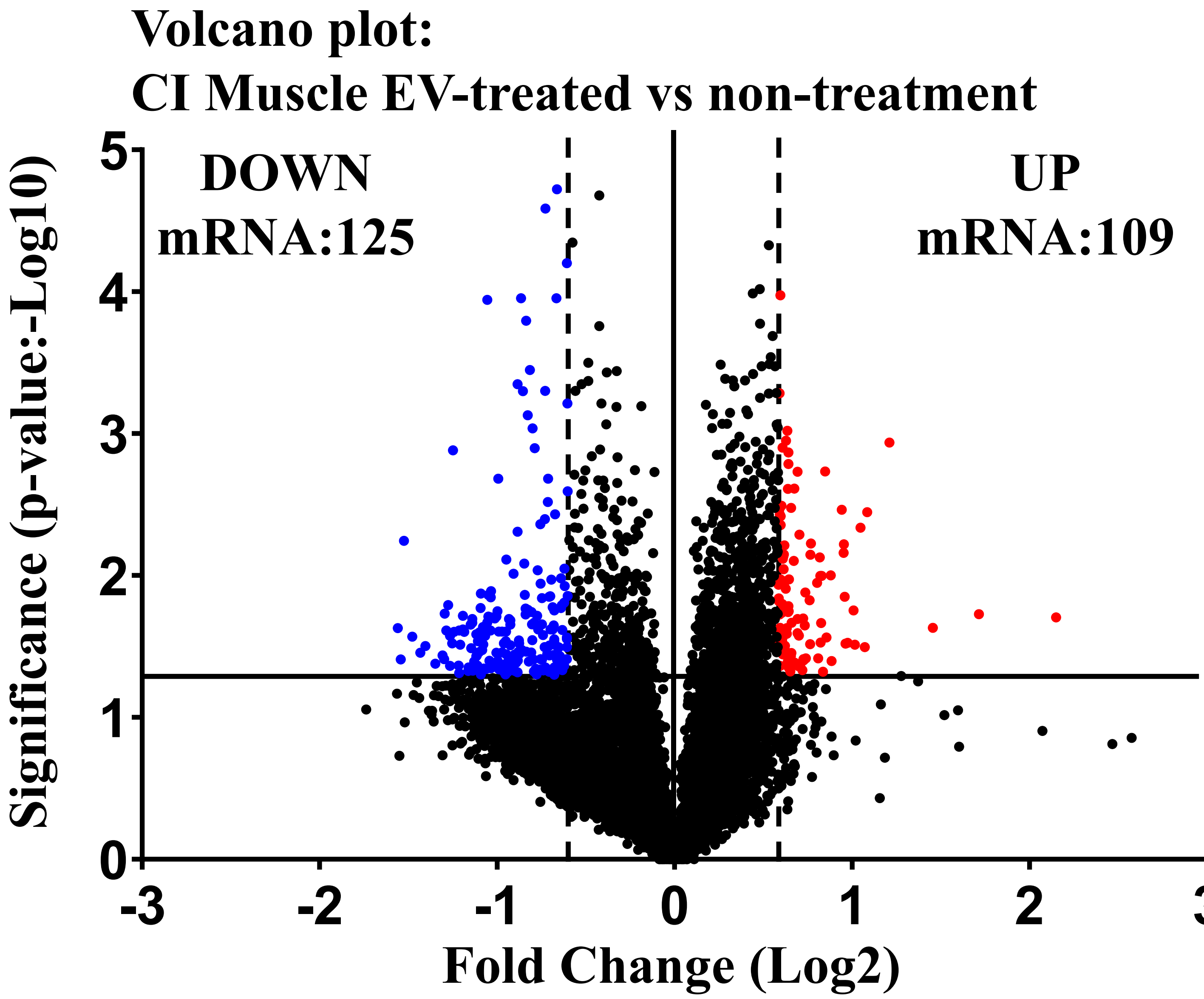

Volcano plots of mRNA-seq transcriptome data displaying the pattern of gene expression values for CI EV- treatment relative to the non- treatment

Supplementary Figure S4

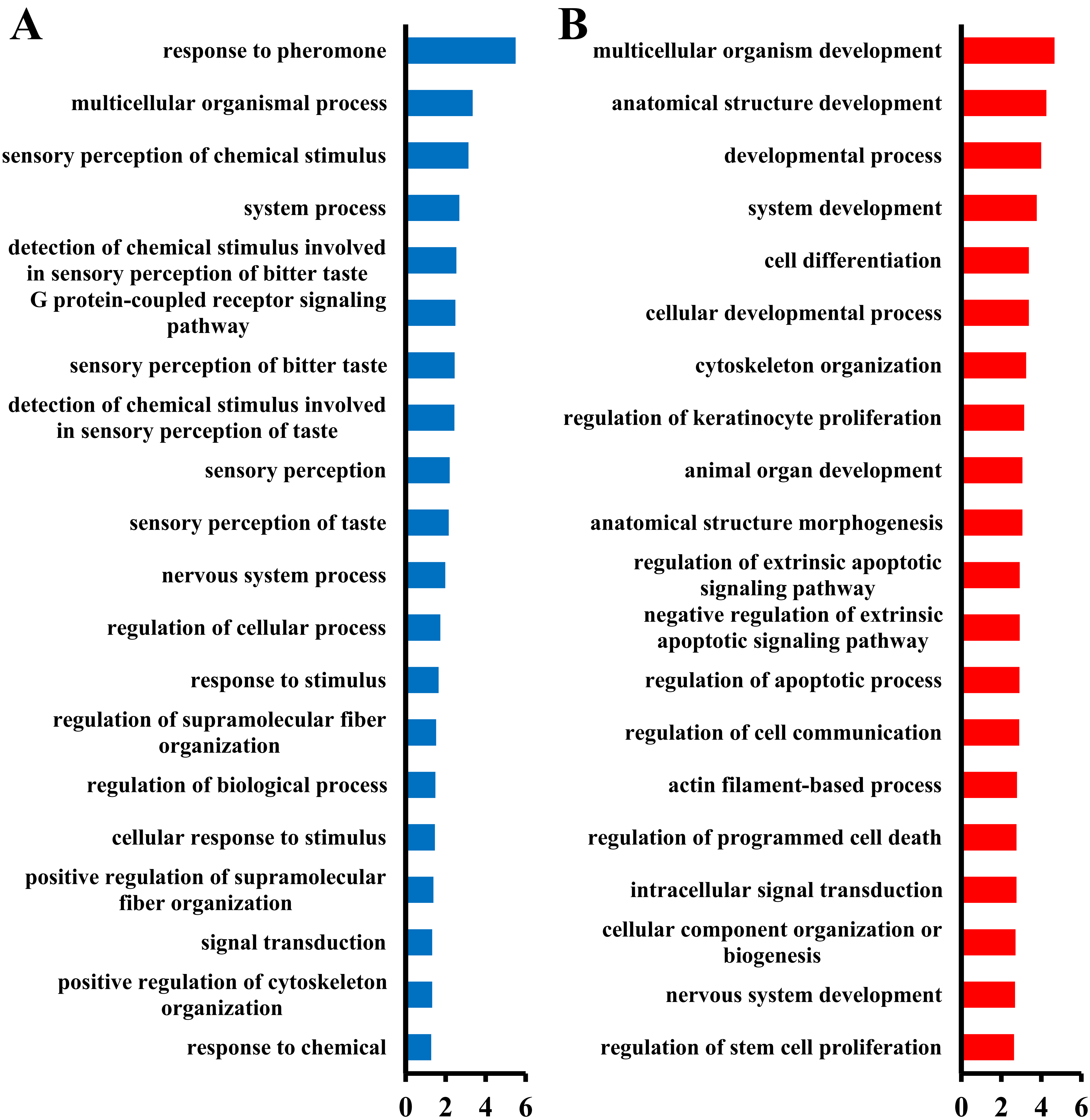

(A) GO enrichment analysis of mRNA decreased in cell CI EV- treatment compared to non- treatment (Top 20 functional GO terms). (B) GO enrichment analysis of mRNA increased in CI EV- treatment compared to non- treatment (Top 20 functional GO terms).
